# Risk of Nonmelanoma Skin Cancers and Parkinson’s Disease—Meta-Analysis and Systematic Review

**DOI:** 10.3390/cancers13040587

**Published:** 2021-02-03

**Authors:** Danuta Krasowska, Agnieszka Gerkowicz, Radosław Mlak, Milena Leziak, Teresa Małecka-Massalska, Dorota Krasowska

**Affiliations:** 1 Department of Dermatology, Venerology and Pediatric Dermatology, Medical University of Lublin, Poland 11 Staszica Street, 20-081 Lublin, Poland; agerkowicz@wp.pl (A.G.); dor.krasowska@gmail.com (D.K.); 2 Department Human Physiology, Medical University of Lublin, Poland 11 Radziwiłłowska Street, 20-080 Lublin, Poland; radoslaw.mlak@gmail.com (R.M.); milena.leziak@wp.pl (M.L.); tmalecka@gmail.com (T.M.-M.)

**Keywords:** Parkinson disease, nonmelanoma skin cancers, meta-analysis, systematic review

## Abstract

**Simple Summary:**

Non-melanoma skin cancers (NMSCs) are the most common cancers among fair-skinned people. It is estimated that 2–3 million new cases of NMSCs are diagnosed globally each year. The risk of development increases with age, just like in the case of Parkinson Disease. Due to the general aging of the population and substantially high medical costs of NMSC therapy, NMSCs and Parkinson’s disease (PD) are becoming an increasing health problem. In this paper, we conduct a meta-analysis and systematic review to evaluate the NMSC risk among patients with PD. This study is the first to focus on the effect of different epidemiologic aspects of NMSCs and PD in detail.

**Abstract:**

Patients with Parkinson’s disease (PD) have an increased risk of melanoma compared with the general population. Considering that Nonmelanoma Skin Cancers (NMSCs) share similar risk factors with melanoma, there is a need to understand a possible connection between PD and NMSCs. The aim of the study was the evaluation of NMSC risk among PD patients via meta-analysis and systematic review. A comprehensive search of PubMed, Scopus, and Web of Science databases was conducted, including studies from January 2000 to April 2020. We identified 16 eligible studies including 140291 PD patients. Upon statistical analysis, a significantly higher risk of developing NMSCs in PD patients was found compared with the control group (odds ratio (OR) = 1.25, 95% CI: 1.17–1.33; *p* < 0.0001). Among all NMSCs, the risk of developing basal cell carcinoma in PD patients was significantly higher (OR = 1.30, 95% confidence interval (CI): 1.15–1.47; *p* < 0.0001), contrary to squamous cell carcinoma. Further analysis revealed a significantly higher risk of developing NMSCs in patients with previously diagnosed PD (OR = 1.26, 95% CI: 1.19–1.33; *p* < 0.0001). Our data suggest the necessity for regular skin examination of PD patients, though further studies are required to explore the mechanisms forming this relationship.

## 1. Introduction

Parkinson’s disease (PD) is a neurodegenerative disease which mainly affects people aged 65 or older [1,2]. For many years, the association between PD and cancers has been the subject of much debate among scientists. On one hand, epidemiological studies show that people with PD have a decreased risk of certain types of cancer compared with the general population [3,4,5,6]. A possible explanation for this unexpected behavior could rest in the opposite mechanisms that lead to the development of those diseases, including increased cell proliferation in malignant processes and cell degeneration in PD [7,8]. Therefore, it is suggested that PD could serve some form of protection against certain cancers [8]. On the other hand, positive associations between PD and melanoma were also confirmed in numerous epidemiological studies [9,10,11]. Recently, it has been reported that patients with PD had a 3.8-fold increased risk of melanoma. Likewise, patients with melanoma had a 4.2-fold increased risk of developing PD [12]. These two diseases demonstrate similarities in biochemical tracts and common genetic pathways [8]. In contrast with melanoma, little is known about the association between PD and non-melanoma skin cancers (NMSCs), including basal cell carcinoma (BCC) and squamous cell carcinoma (SCC). NMSCs are the most common cancers among fair-skinned people [13]. Their occurrence has increased significantly to as much as to 10% per year, and it is estimated that 2–3 million new cases of NMSCs are diagnosed globally each year. It is known that BCCs are more common than SCCs and account for 75% of all NMSC cases [13,14,15]. The risk of NMSC development increases with age, just like demonstrated in the case of PD [8,15]. Due to the general aging of the population and substantially high medical costs of NMSC therapy, NMSCs and PD are becoming an increasing health problem [8,16]. NMSCs share similar risk factors with melanoma, considering that it is very likely that NMSCs may occur more frequently in patients with PD [11]. Therefore, there is a need to understand the possible coexistence between PD and NMSCs. In this paper, we conducted a meta-analysis and systematic review to evaluate the NMSC risk among patients with PD.

## 2. Results

### 2.1. Characteristics of the Studies Included in the Analysis

**Figure 1:**
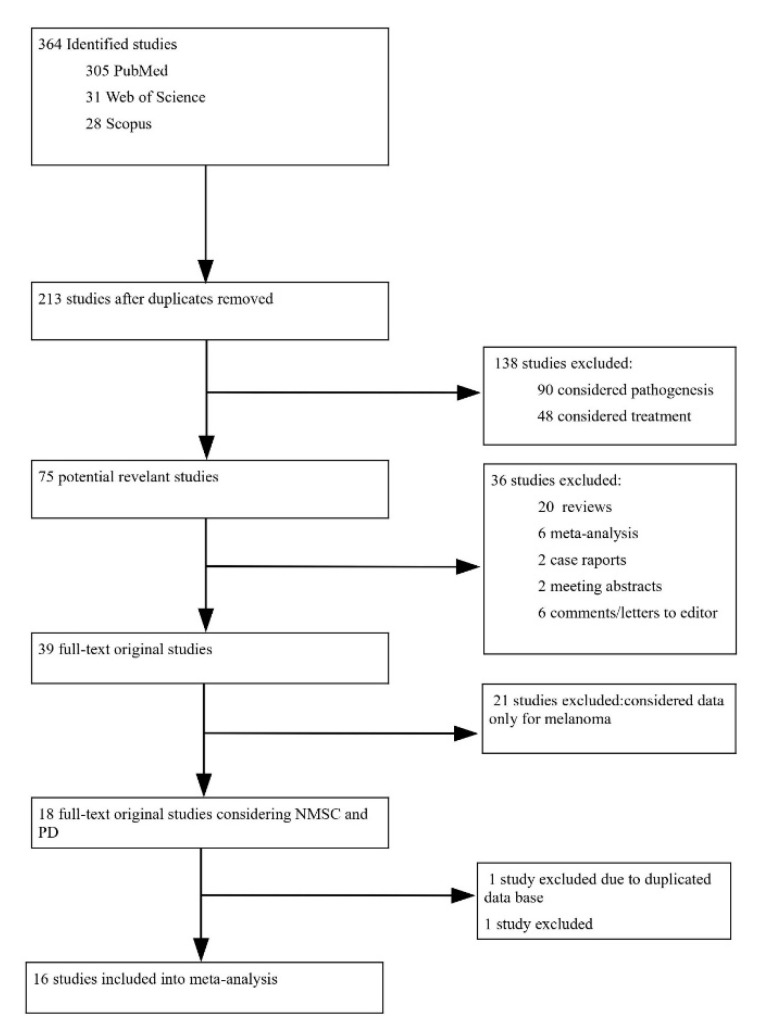
Flow diagram of a paper’s selection process.

We found 364 studies in total. After applying the necessary criteria, the remaining 16 studies were included in the meta-analysis, all of them published between 2002 and 2020. The study selection process is presented in the flow diagram of paper’s selection Figure 1 and supplementary materials Tables S1–S3. The detailed characteristics of the studies which qualified for statistical analysis are presented in Table 1.

### 2.2. Risk of NMSCs in PD patients

**Figure 2:**
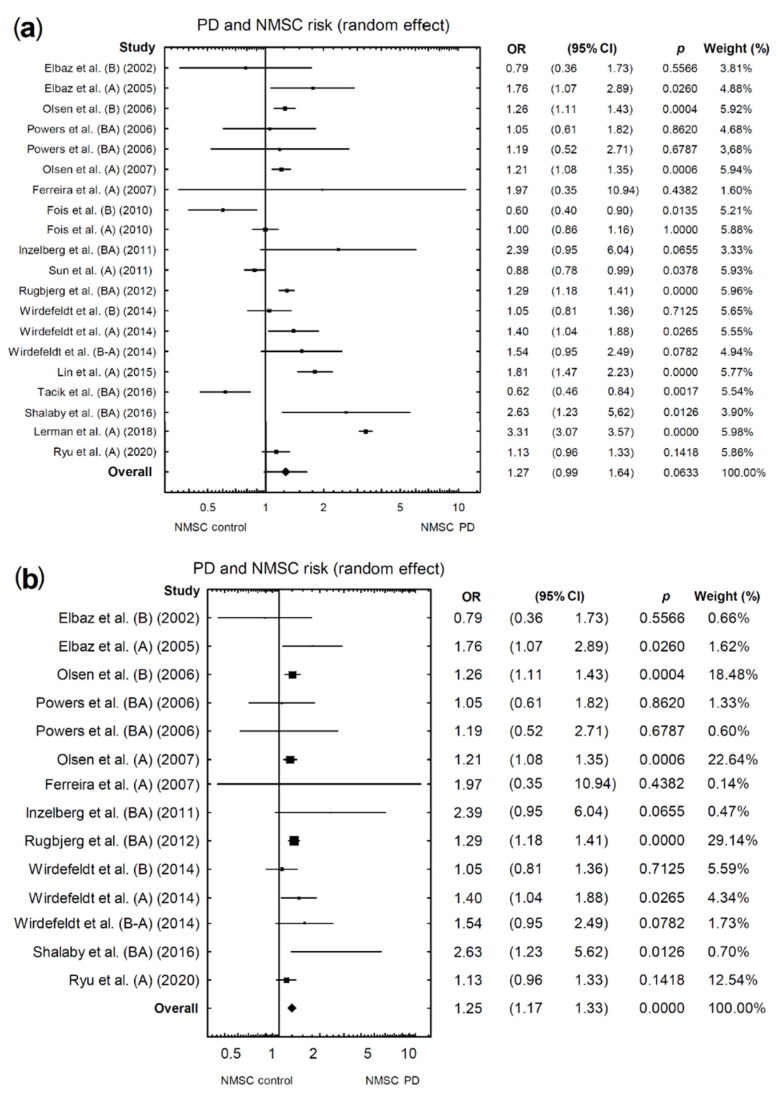
Forest plot of pooled studies of NMSC occurrence in PD patients: (**a**) all studies; (**b**) studies causing high heterogeneity excluded. Each line represents an individual study result, with the width of the horizontal line indicating a 95% confidence interval (CI) and the position of the box representing the point estimate OR (odds ratio) (B—NMSCs before PD diagnosis, A—NMSCs after PD diagnosis, BA—NMSCs diagnosed before or after PD diagnosis, B-A NMSCs diagnosed ±1 year before or after PD diagnosis).

**Figure 3:**
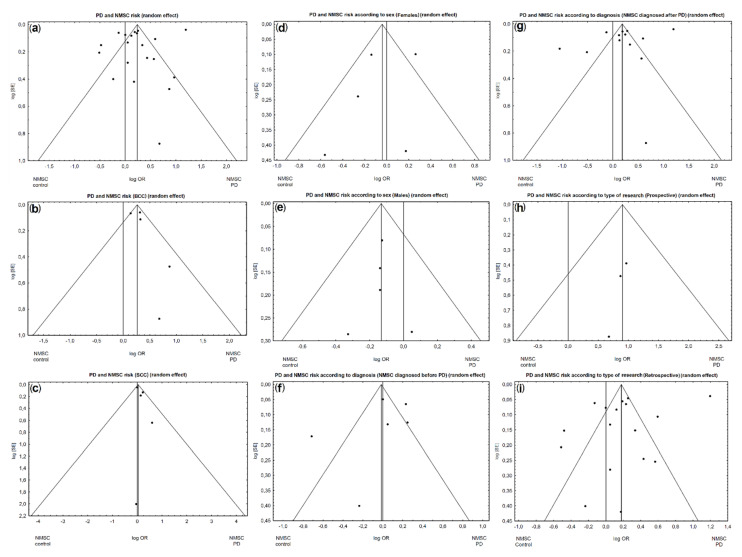
Funnel plot for publication bias in the studies investigating NMSC risk in PD patients: (**a**) all studies; (**b**) BCC; (**c**) squamous cell carcinoma (SCC); (**d**) females; (**e**) males; (**f**) NMSCs diagnosed before PD diagnosis; (**g**) NMSCs diagnosed after PD diagnosis; (**h**) Prospective studies; (**i**) Retrospective studies.

Of the 16 studies included in the analysis, 20 different odds ratio (OR) assessment results were obtained for NMSCs in PD patients. When all available studies were included in the analysis, a marginally higher risk of NMSCs was found in patients with PD (OR = 1.27, 95% confidence interval (CI): 0.99–1.64; *p* = 0.0633; Figure 2a). However, the above analysis showed unacceptable heterogeneity (I2 = 97.05%). Therefore, based on the funnel plot, we found and eliminated studies [3,5,6,11,25] that caused high heterogeneity (Figure 3a). After re-analysis, which accounted for the exclusion of the above studies, we noted a significantly higher risk of developing NMSCs in patients with PD compared with the control group (acceptable heterogeneity: I2 = 11.41%; OR = 1.25, 95% CI: 1.17–1.33; *p* < 0.0001; Figure 2b).

### 2.3. Risk of NMSC Subtypes in PD Patients

In the subgroup analysis by NMSC subtype, we included five studies that provided data separately for BCC and SCC.

#### 2.3.1. BCC

**Figure 4:**
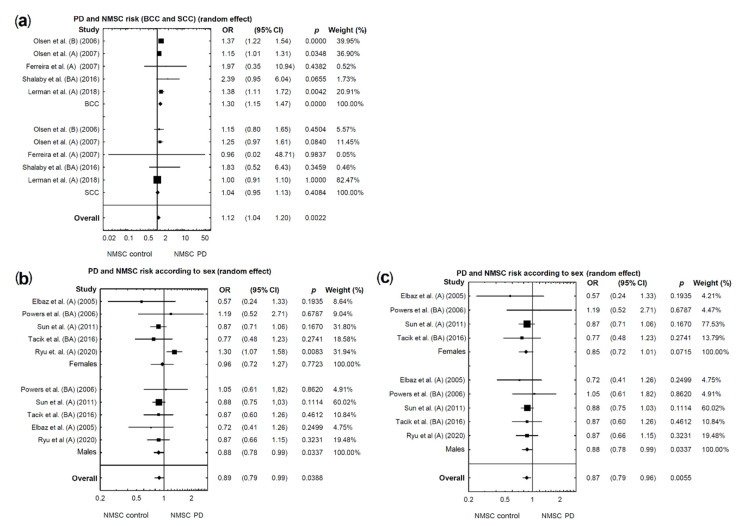
(**a**): Forest plot of pooled studies of BCC and SCC occurrence in PD patients. (**b**) Forest plot of pooled studies of NMSC occurrence in PD patients according to sex. (**c**) Forest plot of pooled studies of NMSC occurrence in PD patients according to sex—studies causing high heterogeneity excluded. Each line represents an individual study result, with the width of the horizontal line indicating a 95% confidence interval (CI) and the position of the box representing the point estimate OR (odds ratio) (B—NMSCs before PD diagnosis, A—NMSCs after PD diagnosis, BA—NMSCs diagnosed before or after PD diagnosis.

A significantly higher risk of BCC was found in patients with PD (acceptable heterogeneity: I2 = 36.69%; Figure 3b; OR = 1.30, 95% CI: 1.15–1.47; *p* < 0.0001; Figure 4a).

#### 2.3.2. SCC

A marginally higher risk of SCC was found in patients with PD (acceptable heterogeneity: I2 = 0%; Figure 3c; OR = 1.04, 95% CI: 0.95–1.13; *p* = 0.4084; Figure 4a).

### 2.4. Sex-Related Risk of NMSC in PD Patients

In the subgroup analysis by sex, we included five studies that provided data separately for male and female patients. A marginally lower risk of NMSCs was found in female and significantly lower in male patients with PD (respectively: OR = 0.96, 95% CI: 0.72–1.27, *p* = 0.7723; OR = 0.89, 95% CI: 0.78–0.99, *p* = 0.0337; Figure 4b). However, the above analysis for females demonstrated unacceptable heterogeneity (I2 = 66.02%; Figure 3d). The same analysis for males demonstrated acceptable heterogeneity (I2 = 0%; Figure 3e).

Therefore, in female patients, based on the funnel plot, we found and eliminated one study which caused high heterogeneity [27]. After re-analysis, a marginally lower risk of developing NMSCs in patients with PD compared with the control group was found (acceptable heterogeneity I2 = 0%; OR = 0.85, 95% CI: 0.72–1.01; *p* = 0.0715; Figure 4c).

### 2.5. Risk of NMSCs in PD Patients Depending on the Order of Diagnosis

**Figure 5:**
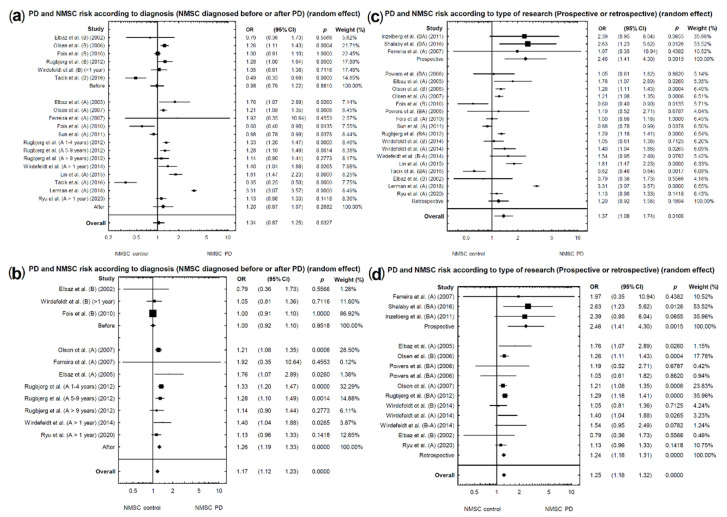
(**a**,**b**) Forest plot of pooled studies of NMSCs diagnosed before or after incidence of PD in patients: (**a**) all studies; (**b**) studies causing high heterogeneity excluded. (**c**,**d**) Forest plot of pooled studies of NMSC incidence in PD patients according to the type of study (prospective or retrospective): (**c**) all studies; (**d**) studies causing high heterogeneity excluded. Each line represents an individual study result, with the width of the horizontal line indicating a 95% confidence interval (CI) and the position of the box representing the point estimate OR (odds ratio) (B—NMSCs before PD diagnosis, B-A NMSCs diagnosed ± 1 year before or after PD diagnosis.

In the subgroup analysis by the order of diagnosis NSMS and PD, we included six studies in which NMSC was diagnosed prior to PD and 13 studies which provided data of NMSC following a diagnosis of PD. A significantly lower risk of NMSCs (diagnosed before PD) was found in patients with PD (OR = 0.98, 95% CI: 0.79–1.22; *p* = 0.8810; Figure 5a), as well as a marginally higher risk of NMSCs (diagnosed after PD) in patients with PD (OR = 1.20, 95% CI: 0.87–1.67; *p* = 0.2682; Figure 5a). In both cases, the above analyses were characterized by unacceptable heterogeneity (respectively: I2 = 84.16%; Figure 3f; I2 = 98.05%; Figure 3g). Studies causing unacceptable heterogeneity were eliminated. From the first group (NMSCs diagnosed before PD diagnosis), the following studies were removed: [19,23,25]. From the second group (NMSCs diagnosed after PD diagnosis), the following studies were removed: [3,5,6,11,25]. After re-analysis, there was no significant effect on the risk of developing NMSCs in patients later diagnosed with PD (NMSCs before PD) (acceptable heterogeneity: I2 = 0%; OR = 1.00, 95% CI: 0.92–1.10; *p* = 0.9518; Figure 5b); also, a significantly higher risk of developing NMSCs in patients with previously diagnosed PD (NMSCs after PD) (acceptable heterogeneity: I2 = 0%; OR = 1.26, 95% CI: 1.19–1.33; *p* < 0.0001; Figure 5b) was found.

### 2.6. Risk of NMSCs in PD Patients Depending on the Type of Research (Prospective or Retrospective)

For prospective studies, a significantly higher risk of NMSCs was found in patients with PD (acceptable heterogeneity: I2 = 0%; Figure 3h; OR = 2.46, 95% CI: 1.41–4.30; *p* = 0.0015; Figure 5c). Analysis of retrospective studies showed a marginally higher risk of NMSCs in patients with PD (unacceptable heterogeneity: I2 = 97.50%; Figure 3i; OR = 1.20, 95% CI: 0.92–1.56; *p* = 0.1894; Figure 5c). Studies causing heterogeneity were eliminated. From the second group (including retrospective and case-control studies), the following studies [3,5,6,11,25] were excluded. No studies were removed from the first group (prospective studies, Figure 5d). After re-analysis, a significant effect with respect to the risk of developing NMSCs in patients with PD (retrospective studies) (acceptable heterogeneity: I2 = 0%; OR = 1.24, 95% CI: 1.18–1.31; *p* <0.0001; Figure 5d) was found.

### 2.7. Evaluation for Publication Bias

The funnel plot charts and Begg’s and Egger’s tests did not reveal any significant publication bias in all created models.

## 3. Discussion

In this paper, we present a meta-analysis and a systematic review which assesses the relationship between PD and NMSCs. To the best of our knowledge, this is the first meta-analysis and systematic review focusing comprehensively on different epidemiological aspects of these two diseases, and this is the novelty of our study. Previous epidemiological studies mainly analyzed the prevalence of melanoma in patients with PD, few also reported data inclusive of other skin cancers [4,6,23,28,29,30,31]. Liu et al. observed no significant association between NMSCs and PD (OR 1.11 (95% CI 0.94–1.30) [31]. Later, Huang et al. found that the risk of NMSCs in PD was slightly higher (OR 1.20, 95% CI 1.11–1.29) than in the general population [30].

Considering the rapid increase in NMSCs annually, we included in this study a comprehensive assessment of literature and data from the previous 20 years, focusing only on NMSCs among PD patients while including the latest data published between January 2020 and April 2020. We then conducted the meta-analysis, which concluded, contrary to previous studies, not only the risk of NMSCs in PD patients, but also the risk of NMSCs depending of the cancer subtype, including BCC and SCC, sex, and the diagnosis order (NMSCs diagnosed before or after PD). In comparison to the previous meta-analyses [30,31], in this paper we excluded one study [32]—the study was performed on the same population as the more recent [27] and we added seven new studies which were not included in the previously mentioned publication [3,6,10,11,25,26,27]. This makes our study the first to analyze the issue of NMSCs among PD patients to such a broad extent.

Our research showed a significantly higher risk of developing NMSCs in patients with PD compared with the control group (OR = 1.25, 95% CI: 1.17–1.33; *p* < 0.0001), which is consistent with previously reported data [30]. The relation between two different diseases in the same individual may be due to common environmental, genetic, and immunological factors. Ultraviolet radiation (UV) is the most important factor contributing to the development of BCC and SCC. These two cancers mainly affect fair-skinned, red-haired, blue-eyed individuals, who have a high predilection to sun-induced skin lesions [9]. It was hypothesized that patients with PD may be more sensitive to UV due to genetically determined lower amounts of eumelanin in the skin [33]. This hypothesis was supported by the result of a prospective study on 38,641 male and 93,661 female patients, which demonstrated that those with red hair are at higher risk of developing PD and the risk of PD decreases in individuals with darker hair [34]. Another study revealed that PD patients had a greater tendency to experience sunburn and found tanning more difficult [33]. It cannot be dismissed that both NMSCs and PD may share similar genetic pathways [8]. One possible link between those two diseases could be the Melanocortin 1 receptor (MCR1) gene variant, which is involved in the pathogenesis of NMSCs. The MCR1 gene is highly polymorphic among Caucasians and some of its variations determine the cancer-prone phenotype of the skin (red hair, blue eyes, and fair skin) [35]. It was demonstrated that individuals with MC1R non-synonymous variants were more prone to have BCC than those without them [36]. The same gene was reported to be connected with PD [37]. It cannot be dismissed that the possible link between PD and NMSCs could lie in a mutation in the epidermal growth factor receptor (EGFR), since EGFR aberrant expression is involved in signaling pathways responsible for cell proliferation, survival, invasion, angiogenesis, and metastasis in many types of cancers, including skin cancers. A high expression rate of EGFR was found in BCC tumoral tissues compared with healthy tissues [38]. What is more, EGFR was reported to interact with several PARK genes [3], in which mutations are responsible for PD.

Interestingly, our meta-analysis revealed that patients with PD have a higher risk of developing BCC (OR = 1.30, 95% CI: 1.15–1.47; *p* < 0.0001) and a marginally higher risk of SCC (OR = 1.04, 95% CI: 0.95–1.13; *p* = 0.4084). This difference may result from the fact that not all studies presented data separately for BCC and SCC [11,19,21,22,26]. Some studies provided only the total number of NMSCs. Another possible explanation for the significantly higher occurrence of BCC is the fact that BCC is the most common type of NMSCs [13,14,15]. The higher risk of developing BCC in the investigated PD patients requires further research. Regarding the diagnosis order, we found a significantly lower risk of NMSCs before the onset of PD (OR = 1, 95% CI: 0.92–1.10; *p* = 0.9518), contrary to the group of patients where NMSCs followed the diagnosis of PD, in which case the risk was significantly higher (OR = 1.28, 95% CI: 1.20–1.36; *p* < 0.0001). This confirms the hypothesis that PD patients may be more prone to the development of sun-induced skin lesions. Therefore, the onset of NMSC after diagnosis of PD could result from disease-specific susceptibility or the photocarcinogenic effect of anti-parkinsonian drugs [9]. However, further research including prospective studies and assessment of anti-parkinsonian drugs on the development of NMSCs are required to confirm or reject this hypothesis [11]. Elbaz et al. suggested that more frequent diagnoses of NMSCs after the onset of PD may result from the increased frequency of medical consultations among patients in this group [18].

In our study, sex-specific analysis revealed a significantly lower risk of NMSC in male patients with PD (OR = 0.88, 95% CI: 0.79–0.99; *p* = 0.0388) and a marginally lower risk in female patients. The results are intriguing since NMSCs are more common in males, which may be explained partly by more extensive sun exposure due to working outdoors more frequently [39]. However, Kenborg et al. revealed that outdoor workers had a lower risk of PD development than indoor workers [40]. Therefore, it cannot be excluded that observations in our study correlations may result from different sun exposure habits among PD patients. Sex-related susceptibility on NMSC in PD patients requires further studies. In particular, the significantly higher risk of developing NMSCs was observed for both study types. In the case of prospective studies, we indicated a considerably strong relation between the two diseases (OR = 2.46, 95% CI: 1.41–4.30; *p* = 0.0015). Also, analysis of retrospective studies showed a significant effect on the risk of developing NMSCs in patients with PD (OR = 1.26, 95% CI: 1.19–1.33; *p* < 0.0001). The probable difference between ORs may be explained by the fact that the retrospective studies based on medical records of patients could not have been accurately followed up.

This study has some limitations. Not all studies included in our meta-analysis provided the detailed data concerning the control group. All analyzed studies reported results adjusted for age (the only exceptions were: three studies [19,24,26] in which the control group were matched by age). Among all studies included in this meta-analysis, five studies [11,19,21,22,26] provided detailed information about BCC and SCC. The remaining studies provided a total number of all NMSC cases without specifying types of skin cancers. We cannot dismiss the fact that the studies not only analyzed keratinocyte carcinomas, which are the most common types of NMSCs, but may also include rare types of NMSC cancers.

## 4. Materials and Methods

### 4.1. Search Strategy

We conducted a comprehensive literature search of PubMed, Scopus, and Web of Science databases. We searched for original observational studies reporting associations between NMSCs and Parkinson’s disease, dated from January 2000 to April 2020. We used medical subject heading (MeSH) terms, such as: “PD,” “Carcinoma, Squamous Cell,” “Carcinoma, Basal Cell”, “skin neoplasm”, and text terms, including: “Parkinson disease*”, “non-melanoma skin cancer*”, “basal cell carcinoma*”, “squamous cell carcinoma*”, “skin cancer”, and “keratinocyte carcinoma*”. The search was optimized for each database, taking into consideration differences in search syntaxes. The search was restricted to studies involving humans and published in English.

### 4.2. Eligibility Criteria

Studies were evaluated independently by three co-authors. The potential studies were evaluated by screening titles and abstracts. The definitive inclusion of studies was made after reviewing the full-text articles. Studies were included in the analysis if they reported a number of patients diagnosed with both NMSCs, PD and risk estimates. The exclusion criteria were as follows: reviews, case reports, meeting abstracts, comments, letters to editor and studies with insufficient data for the meta-analysis.

### 4.3. Data Extraction

Extracted data from eligible studies included: name of first author; date of the study; the sample of the studied population; the patients’ characteristics, including age, sex, and country; diagnostic criteria for PD and NMSCs; subtype of non-melanoma skin cancer, type of research and risk estimates, including: odds radio (OR), hazard ratio (HR), or standardized incidence ratio (SIR), with 95% confidence intervals or data that enabled their calculation.

### 4.4. Statistical Analysis

Data analysis, including comparison of the occurrence and OR of NMSC in patients with PD, was performed with the use of the Statistica software (v. 13 PL). OR and the corresponding 95% confidence intervals (CI) were calculated in patients assigned to the NMSC group, compared with patients assigned to the non-NMSCs group and taking into account the presence or absence of PD in the same study. OR > 1.0 indicates a higher risk or more frequent occurrence of NMSCs in patients with PD compared with controls. Random models were used to calculate the OR. The selection of studies included in subsequent stages of the analysis took into account data on their heterogeneity. The Q test was used to assess heterogeneity, while I2 was used to assess inconsistency. We evaluated the potential bias of publications using Begg’s and Egger’s tests and funnel plot charts. When significant heterogeneity was observed in the analysis (*p* < 0.05 or I2 > 50%) and the withdrawal of data from the publication illustrated by funnel plot charts, we performed a further selection of studies. Sensitivity analysis was performed by removing one study at a time to determine if the results would affect a single study, especially in the face of suspicious results or significant heterogeneity. Subgroup analysis was performed by NMSC subtype (BCC or SCC), diagnosis order (NMSCs before/after PD), sex, and study type (prospective or retrospective). A two-sided *p*-value < 0.05 was considered statistically significant.

## 5. Conclusions

To sum up, this study was the first to focus in a comprehensive manner on different epidemiologic aspects of NMSCs and PD, including subtype of cancer, sex, the temporal relationship between both diseases and study type. We found a higher risk of NMSCs in PD patients. Our data suggest the necessity for regular skin examinations of PD patients. Further studies are required to evaluate the connection between those two diseases.

## Figures and Tables

**Table 1:** Characteristics of the studies qualified for statistical analysis. PD—Parkinson disease, NMSCs—nonmelanoma skin cancers, BCC—basal cell carcinoma, OR—odds ratio, CI—confidence interval.

No.	Authors	Year of Publication	Diagnosis before or after PD	Type of Research	PD-NMSCs/ PD *n*, (%)	OR	95%CI	Country	Sex	PD Diagnosis	Cancer Diagnosis
1	Elbaz [17]	2002	Before	Retrospective	13/196 (2.88%)	0.79	0.36–1.73	The United States	Both	Medical records	Medical records
2	Elbaz [18]	2005	After	Retrospective	39/196 (6.63%)	1.76	1.07–2.89	The United States	Both	Medical records	Medical records
3	Olsen [19]	2006	Before	Population-based case-control study	343/8090 (4.24%)	1.26	1.11–1.43	Dennmark	Both	Medical records	Medical records
4	Powers [20]	2006	Both	Case-control study	39/352 (19.90%)	1.05	0.61–1.82	The United States	Men	Medical records	Self-reported questionnaire
5	Powers [20]	2006	Both	Case-control study		1.19	0.53–2.71	The United States	Women	Medical records	Self-reported questionnaire
6	Olsen [21]	2007	After	Retrospective	328/14088 (2.33%)	1.21	1.09–1.35	Dennmark	Both	Medical records	Medical records
7	Ferreira [22]	2007	After	Prospective	4/150 (2.67%)	1.97	0.36–10.94	Portugal	Both	Neurologist	Dermatologist /pathology reports
8	Fois [5]	2010	Before	Retrospective	168/4355 (3.85%)	1	0.80–1.10	England	Both	Medical records	Medical records
9	Fois [5]	2010	After	Retrospective	17/4355 (0.39%)	0.6	0.30–0.90	England	Both	Medical records	Medical records
10	Inzelberg [10]	2011	Both (only BCC)	Prospective	8/1395 (0.57%)	2.39	0.95–6.04	Israel	Both	Neurologist	Dermatologist/ pathology report
11	Sun [6]	2011	After	Retrospective	-	0.88	0.78–0.99	Taiwan	Both	Medical records	Medical records
12	Rugbjerg [23]	2012	Both	Retrospective	586/20343 (25.00%)	1.29	1.18–1.39	Dennmark	Both	Medical records	Medical records
13	Wirdefeldt [24]	2014	Before (>1 year)	Retrospective	172/11786 (1.46%)	1.05	0.82–1.36	Sweden	Both	Medical records	Medical records (clinical/pathological)
14	Wirdefeldt [24]	2014	After (>1 year)	Retrospective		1.4	1.04–1.88	Sweden	Both	Medical record	Medical records (clinical/pathological)
15	Wirdefeldt [24]	2014	±1 year before or after	Retrospective		1.54	0.96–2.49	Sweden	Both	Medical records	Medical records (clinical/pathological)
16	Lin [3]	2015	After	Retrospective	-	1.81	1.46–2.23	Taiwan	Both	Medical records	Medical records histological report
17	Tacik [25]	2016	Both	Retrospective	155/971 (11.08%)	0.62	0.45–0.84	The United States	Both	Movement disorder specialist	Medical records
18	Shalaby [26]	2016	Both	Prospective	27/108 (9.54%)	2.63	1.24–5.62	The United States	Both	Neurologist	Dermatologist/Pathology reports
19	Lerman [11]	2018	After	Retrospective	737/7727 (0.68%)	3.31	3.06–3.57	Israel	Both	Medical records	Pathology reports
20	Ryu [27]	2020	After (>1 year)	Retrospective	186/70730 (0.26%)	1.13	0.96–1.32	Korea	Both	Medical records	Medical records

## Data Availability

This article is an open access article distributed under the terms and conditions of the Creative Commons Attribution(CC BY) license (https://creativecommons.org/licences/by/4.0/).

## Supplementary Materials

The following are available online at https://www.mdpi.com/2072-6694/13/4/587/s1: Detailed search strategy, including Table S1: Selection criteria, Table S2: Search results after removing studies considering only melanoma, Table S3: Studies qualified for meta-analysis.
