# Supplementary material for: Risk of Nonmelanoma Skin Cancers and Parkinson’s Disease—Meta-Analysis and Systematic Review
Source: Cancers (Basel). 2021 Feb 3;13(4):587. doi: 10.3390/cancers13040587 (PMC7913207; doi:10.3390/cancers13040587)
Supplement: Supplementary file 1 [file cancers-13-00587-s001.pdf]

Supporting information file

Title: *Risk of Nonmelanoma Skin Cancers and Parkinson's Disease – Meta-Analysis and Systematic Review*

Authors: Danuta Krasowska[a], Agnieszka Gerkowicz[a], Radosław Mlak[b,] Milena Leziak[b], Teresa Małecka-Massalska[b], Dorota Krasowska[a]

Corresponding Author: Danuta Krasowska

Search strategy

We used medical subject heading (MeSH) terms, such as: "PD," "Carcinoma, Squamous Cell," " Carcinoma, Basal Cell," "skin neoplasm," and text terms, including: "Parkinson disease*," "non-melanoma skin cancer*," "basal cell carcinoma*," "squamous cell carcinoma*," "skin cancer," and "keratinocyte carcinoma*." The search was optimized for each database, taking into consideration differences in the search syntaxes. The search was restricted to studies involving humans and published in English.

Inclusion of studies was made after reviewing the full-text articles. Studies were included in the analysis if they reported a number of patients diagnosed with both NMSCs and PD, and risk estimates.

The exclusion criteria were as follows: reviews, case reports, meeting abstracts, comments, letters to editor and studies with insufficient data for the meta-analysis (lack of OR, HR, SIR, lack of population count or other data which would allow independent calculation of above risk indicators)

(1)

Table S1. Selection criteria.

Number	Selection process	Number of all studies qualified for further selection	Number of studies exluded
1	All results from January2000 till April 2020	364	-
2	Remove duplicates	213	151
3	Remove studies considering treatment	165	48
4	Remove studies considering pathogenesis	75	90
5	Remove review	55	20
6	Remove meta-analysis	49	6
7	Remove abstract meetings	47	2
8	Remove case reports	45	2
9	Remove comments/ letters to editors	39	6
10	Remove studies considering only melanoma	18	21
11	Leave studies considering NMSC that full fill inclusion criteria	16	2

Table S2. Search results after removing studies considering only melanoma.

1. Bertoni JM, Arlette JP, Fernandez HH, Fitzer-Attas C, Frei K, Hassan MN, Isaacson SH, Lew MF, Molho E, Ondo WG, Phillips TJ, Singer C, Sutton JP, Wolf JE Jr; North American Parkinson's and Melanoma Survey Investigators. Increased melanoma risk in Parkinson disease: a prospective clinicopathological study. Arch Neurol. 2010 Mar;67(3):347-52. doi: 10.1001/archneurol.2010.1. PubMed PMID: 20212233.
2. Elbaz A, Peterson BJ, Yang P, Van Gerpen JA, Bower JH, Maraganore DM, McDonnell SK, Ahlskog JE, Rocca WA. Nonfatal cancer preceding Parkinson's disease: a case-control study. Epidemiology. 2002 Mar;13(2):157-64. PubMed PMID: 11880756.
3. Elbaz, A., Peterson, B.J., Bower, J.H., (...), Ahlskog, J.E., Rocca, W.A.2005 Movement Disorders 20(6), pp. 719-725 Risk of cancer after the diagnosis of Parkinson's disease: A historical cohort study Atypical cancer pattern in patients with Parkinson's disease Olsen 2005
4. Ferreira J, Silva JM, Freire R, Pignatelli J, Guedes LC, Feijó A, Rosa MM, Coelho M, Costa J, Noronha A, Hewett R, Gomes AM, de Castro JL, Rascol O, Sampaio C. Skin cancers and precancerous lesions in Parkinson's disease patients. Mov Disord. 2007 Jul 30;22(10):1471-5. PubMed PMID: 17516496.
5. Fois AF, Wotton CJ, Yeates D, Turner MR, Goldacre MJ. Cancer in patients with motor neuron disease, multiple sclerosis and Parkinson's disease: Record linkage studies. J Neurol Neurosurg Psychiatry 2010;81:215–21
6. Inzelberg R, Rabey JM, Melamed E, Djaldetti R, Reches A, Badarny S, Hassin-Baer S, Cohen O, Trau H, Aharon-Peretz J, Milo R, Schwartz M, Huberman M, Gilead L, Barchana M, Liphshiz I, Fitzer-Attas C, Giladi N. High prevalence of malignant melanoma in Israeli patients with Parkinson's disease. J Neural Transm (Vienna). 2011 Aug;118(8):1199-207. doi: 10.1007/s00702-011-0580-2. Epub 2011 Feb 5. PubMed PMID: 21298300.
7. Lerman S, Amichai B, Weinstein G, Shalev V, Chodick G. Parkinson's Disease, Melanoma, and Keratinocyte Carcinoma: A Population-Based Study. Neuroepidemiology. 2018;50(3-4):168-173. doi: 10.1159/000487855. Epub 2018 Mar 22. PubMed PMID: 29566384.
8. Lin PY, Chang SN, Hsiao TH, Huang BT, Lin CH, Yang PC. Association Between Parkinson Disease and Risk of Cancer in Taiwan. JAMA Oncol. 2015 Aug;1(5):633-40. doi: 10.1001/jamaoncol.2015.1752. PubMed PMID: 26181771.
9. Olsen JH, Friis S, Frederiksen K, McLaughlin JK, Mellemkjaer L, Møller H. Atypical cancer pattern in patients with Parkinson's disease. Br J Cancer. 2005 Jan 17;92(1):201-5. PubMed PMID: 15583688; PubMed Central PMCID: PMC2361753.
10. Olsen JH, Friis S, Frederiksen K. Malignant melanoma and other types of cancer preceding Parkinson disease. Epidemiology. 2006 Sep;17(5):582-7. PubMed PMID: 16837822.
11. Olsen JH, Tangerud K, Wermuth L, Frederiksen K, Friis S. Treatment with levodopa and risk for malignant melanoma. Mov Disord. 2007 Jul 15;22(9):1252-7. PubMed PMID: 17534943.
12. Powers KM, Smith-Weller T, Franklin GM, Longstreth WT, Swanson PD, Checkoway H. Diabetes, smoking, and other medical conditions in relation to Parkinson's disease risk. Park Relat Disord 2006;12:185–9doi:10.1016/j.parkreldis.2005.09.004.
13. Rugbjerg K, Friis S, Lassen CF, Ritz B, Olsen JH. Malignant melanoma, breast cancer and other cancers in patients with Parkinson's disease. Int J Cancer. 2012 Oct 15;131(8):1904-11. doi: 10.1002/ijc.27443. Epub 2012 Mar 22. PubMed PMID: 22278152; PubMed Central PMCID: PMC3636769.
14. Ryu HJ, Park J-H, Choi M, et al. Parkinson's Disease and Skin Cancer Risk: A Nationwide Population-Based Cohort Study in Korea. J Eur Acad Dermatology Venereol 2020.
15. Shalaby SY, Louis ED. Increased Odds of Melanoma: Parkinson's Disease, Essential Tremor, Dystonia versus Controls. Neuroepidemiology. 2016;46(2):128-36. doi: 10.1159/000443794. Epub 2016 Jan 28. PubMed PMID: 26820576; PubMed Central PMCID: PMC5473151.
16. Sun LM, Liang JA, Chang SN, Sung FC, Muo CH, Kao CH. Analysis of Parkinson's disease and subsequent cancer risk in Taiwan: a nationwide population-based cohort study. Neuroepidemiology. 2011;37(2):114-9. doi: 10.1159/000331489. Epub 2011 Oct 7. PubMed PMID: 21986194.
17. Tacik P, Curry S, Fujioka S, Strongosky A, Uitti RJ, van Gerpen JA, Diehl NN, Heckman MG, Wszolek ZK. Cancer in Parkinson's disease. Parkinsonism Relat Disord. 2016 Oct;31:28-33. doi: 10.1016/j.parkreldis.2016.06.014. Epub 2016 Jun 20. PubMed PMID: 27372241; PubMed Central PMCID: PMC5048511.
18. Wirdefeldt K, Weibull CE, Chen H, Kamel F, Lundholm C, Fang F, Ye W. Parkinson's disease and cancer: A register-based family study. Am J Epidemiol. 2014 Jan 1;179(1):85-94. doi: 10.1093/aje/kwt232. Epub 2013 Oct 18. PubMed PMID: 24142916; PubMed Central PMCID: PMC3864714.

Excluded studies:

1. Olsen JH, Friis S, Frederiksen K, McLaughlin JK, Mellemkjaer L, Møller H. Atypical cancer pattern in patients with Parkinson's disease. Br J Cancer. 2005 Jan;92(1):201–5.
2. Bertoni JM, Arlette JP, Fernandez HH, Fitzer-Attas C, Frei K, Hassan MN, Isaacson SH, Lew MF, Molho E, Ondo WG, Phillips TJ, Singer C, Sutton JP, Wolf JE Jr; North American Parkinson's and Melanoma Survey Investigators. Increased melanoma risk in Parkinson disease: a prospective clinicopathological study. Arch Neurol. 2010 Mar;67(3):347-52. doi: 10.1001/archneurol.2010.1. PubMed PMID: 20212233.

The study by (Olsen et al 2005) was performed on the same population as the more recent. Olsen et all 2006.). Therefore for further analysis we included only the more recent study to avoid data duplication

The study by (Bertoni et al, 2010) was excluded since it not provided any of the following: the result of the estimation NMSC risk in PD patients (e.g. OR, HR, SIR) nor the number of control (including without NMSC or not PD), which would allow the independent calculation of the above risk indicators

Table S3. Studies qualified for meta-analysis.

1. Elbaz A, Peterson BJ, Yang P, Van Gerpen JA, Bower JH, Maraganore DM, McDonnell SK, Ahlskog JE, Rocca WA. Nonfatal cancer preceding Parkinson's disease: a case-control study. Epidemiology. 2002 Mar;13(2):157-64. PubMed PMID: 11880756.
2. Elbaz, A., Peterson, B.J., Bower, J.H., (...), Ahlskog, J.E., Rocca, W.A.2005 Movement Disorders 20(6), pp. 719-725 Risk of cancer after the diagnosis of Parkinson's disease: A historical cohort study Atypical cancer pattern in patients with Parkinson's disease Olsen 2005
3. Ferreira J, Silva JM, Freire R, Pignatelli J, Guedes LC, Feijó A, Rosa MM, Coelho M, Costa J, Noronha A, Hewett R, Gomes AM, de Castro JL, Rascol O, Sampaio C. Skin cancers and precancerous lesions in Parkinson's disease patients. Mov Disord. 2007 Jul 30;22(10):1471-5. PubMed PMID: 17516496.
4. Fois AF, Wotton CJ, Yeates D, Turner MR, Goldacre MJ. Cancer in patients with motor neuron disease, multiple sclerosis and Parkinson's disease: Record linkage studies. J Neurol Neurosurg Psychiatry 2010;81:215–21
5. Inzelberg R, Rabey JM, Melamed E, Djaldetti R, Reches A, Badarny S, Hassin-Baer S, Cohen O, Trau H, Aharon-Peretz J, Milo R, Schwartz M, Huberman M, Gilead L, Barchana M, Liphshiz I, Fitzer-Attas C, Giladi N. High prevalence of malignant melanoma in Israeli patients with Parkinson's disease. J Neural Transm (Vienna). 2011 Aug;118(8):1199-207. doi: 10.1007/s00702-011-0580-2. Epub 2011 Feb 5. PubMed PMID: 21298300.
6. Lerman S, Amichai B, Weinstein G, Shalev V, Chodick G. Parkinson's Disease, Melanoma, and Keratinocyte Carcinoma: A Population-Based Study. Neuroepidemiology. 2018;50(3-4):168-173. doi: 10.1159/000487855. Epub 2018 Mar 22. PubMed PMID: 29566384.
7. Lin PY, Chang SN, Hsiao TH, Huang BT, Lin CH, Yang PC. Association Between Parkinson Disease and Risk of Cancer in Taiwan. JAMA Oncol. 2015 Aug;1(5):633-40. doi: 10.1001/jamaoncol.2015.1752. PubMed PMID: 26181771.
8. Olsen JH, Friis S, Frederiksen K. Malignant melanoma and other types of cancer preceding Parkinson disease. Epidemiology. 2006 Sep;17(5):582-7. PubMed PMID: 16837822.
9. Olsen JH, Tangerud K, Wermuth L, Frederiksen K, Friis S. Treatment with levodopa and risk for malignant melanoma. Mov Disord. 2007 Jul 15;22(9):1252-7. PubMed PMID: 17534943.

10. Powers KM, Smith-Weller T, Franklin GM, Longstreth WT, Swanson PD, Checkoway H. Diabetes, smoking, and other medical conditions in relation to Parkinson's disease risk. Park Relat Disord 2006;12:185–9doi:10.1016/j.parkreldis.2005.09.004.

11. Rugbjerg K, Friis S, Lassen CF, Ritz B, Olsen JH. Malignant melanoma, breast cancer and other cancers in patients with Parkinson's disease. Int J Cancer. 2012 Oct 15;131(8):1904-11. doi: 10.1002/ijc.27443. Epub 2012 Mar 22. PubMed PMID: 22278152; PubMed Central PMCID: PMC3636769.

12. Ryu HJ, Park J-H, Choi M, et al. Parkinson's Disease and Skin Cancer Risk: A Nationwide Population-Based Cohort Study in Korea. J Eur Acad Dermatology Venereol 2020.

13. Shalaby SY, Louis ED. Increased Odds of Melanoma: Parkinson's Disease, Essential Tremor, Dystonia versus Controls. Neuroepidemiology. 2016;46(2):128-36. doi: 10.1159/000443794. Epub 2016 Jan 28. PubMed PMID: 26820576; PubMed Central PMCID: PMC5473151.

14. Sun LM, Liang JA, Chang SN, Sung FC, Muo CH, Kao CH. Analysis of Parkinson's disease and subsequent cancer risk in Taiwan: a nationwide population-based cohort study. Neuroepidemiology. 2011;37(2):114-9. doi: 10.1159/000331489. Epub 2011 Oct 7. PubMed PMID: 21986194.

15. Tacik P, Curry S, Fujioka S, Strongosky A, Uitti RJ, van Gerpen JA, Diehl NN, Heckman MG, Wszolek ZK. Cancer in Parkinson's disease. Parkinsonism Relat Disord. 2016 Oct;31:28-33. doi: 10.1016/j.parkreldis.2016.06.014. Epub 2016 Jun 20. PubMed PMID: 27372241; PubMed Central PMCID: PMC5048511.

16. Wirdefeldt K, Weibull CE, Chen H, Kamel F, Lundholm C, Fang F, Ye W. Parkinson's disease and cancer: A register-based family study. Am J Epidemiol. 2014 Jan 1;179(1):85-94. doi: 10.1093/aje/kwt232. Epub 2013 Oct 18. PubMed PMID: 24142916; PubMed Central PMCID: PMC3864714.
